# Effects of Oral Folic Acid and Iron Tablets Intake on the Prevalence and Severity of Anemia in Pregnant Women in a Public Sector Hospital in Delhi

**DOI:** 10.7759/cureus.69041

**Published:** 2024-09-09

**Authors:** Nishal Sharma, Monika Gupta, Jugal Kishore, Himal Singla, Rohini Dayma, Jai Bhagwan Sharma

**Affiliations:** 1 Community Medicine, Vardhman Mahavir Medical College and Safdarjung Hospital, New Delhi, IND; 2 Obstetrics and Gynaecology, Vardhman Mahavir Medical College and Safdarjung Hospital, New Delhi, IND; 3 Obstetrics and Gynaecology, All India Institute of Medical Sciences, New Delhi, IND

**Keywords:** anemia, oral folic acid, oral iron, pregnancy, prevalence, tea

## Abstract

Objective: To study the effects of oral folic acid, iron tablets, and tea consumption on the prevalence and severity of anemia in pregnancy.

Methods: This cross-sectional study was conducted on 430 women who fulfilled the eligibility criteria in the second and third trimesters of pregnancy attending the antenatal clinic of a public sector hospital in Delhi.

Results: The mean age, parity, BMI, and gestation were 26.2 ± 4.5 years, 1.8 ± 1.2, 22.5 ± 4.5 kg/m^2^, and 32.2 ± 3.4 weeks, respectively. Out of 430 patients, the prevalence of anemia was found in 210 (48.84%) patients, with mild anemia in 111 (25.81%), moderate in 68 (15.81%), severe in 30 (6.98%), and very severe in one (0.24%) patient. Significantly more women (97, 46.19%) in the anemia group did not take oral folic acid tablets as compared to the normal hemoglobin (Hb) group (83, 37.72%) (p = 0.04). Similarly, significantly more (103, 49.04%) women in the anemia group did not take oral iron tablets as compared to the normal Hb group (19, 8.63%) (p = 0.02) with even more patients being in the severe anemia group (29, 93.55%) (p = 0.001). Intake of two or more cups of tea per day was a significant risk factor for anemia, with 147 (70%) anemic vs. 134 (60.9%) with normal Hb (p = 0.05).

Conclusion: The prevalence of anemia during pregnancy was found to be high in 210 (48.84%) patients. Non-intake of oral folic acid and iron tablets and consumption of two or more cups of tea were significant risk factors for anemia in pregnancy.

## Introduction

Anemia is the most common disease affecting almost 1.5 billion people worldwide, with prevalence being higher in Asia and Africa [1,2]. Iron deficiency anemia accounts for 50% of cases of anemia [1,2]. Pregnant women are especially vulnerable to anemia with an overall prevalence of anemia during pregnancy in India being 52.2% in the National Family Health Survey-5 (NFHS-5) (2019-2021), which is more than 50.4% of NFHS-4 (2016-2018) [3] and is more than 38.2% of global prevalence [4]. A hemoglobin (Hb) concentration of less than 11 g/dl or a hematocrit of less than 33% is taken as the definition of anemia in pregnancy [4-6]. Nutritional deficiency (most commonly iron, followed by folate and vitamin B12) is the most common cause of anemia, followed by hemoglobinopathies, infections, worm infestations, malignancies, and chronic diseases [4-7].

Anemia during pregnancy has adverse maternal and perinatal outcomes like premature birth, fetal growth restriction, placental problems, decreased maternal reserves and decreased breast milk production, and neurocognitive and mental health consequences in the babies [2,4,8,9]. Hence, prevention and timely diagnosis and management can help in optimum maternal and perinatal outcomes [10]. The common etiology of anemia in pregnancy is iron deficiency being responsible for 50-60% of cases due to reduced iron available as a result of insufficient dietary iron intake, poor absorption, and increased losses from vomiting or bleeding, further compounded by increased iron demands in pregnancy [5,6]. The WHO and the Government of India recommend routine iron supplementation in doses of 60 mg elemental iron and 500 µg folic acid to all pregnant women if their Hb is more than 11 g/dl and in therapeutic doses (120 mg elemental iron) if there is anemia in pregnancy [1,5,11].

The present study was conducted to see the prevalence and severity of anemia in pregnancy and to see the effect of oral iron and folic acid tablets on the prevalence of anemia along with other factors like frequency and number of cups of tea in a public hospital in Delhi.

## Materials and methods

Study population

It was a cross-sectional study conducted on 430 willing pregnant women in the second and third trimesters of pregnancy attending the antenatal clinic of a public sector tertiary referral hospital from January 2023 to September 2023.

**Figure 1:**
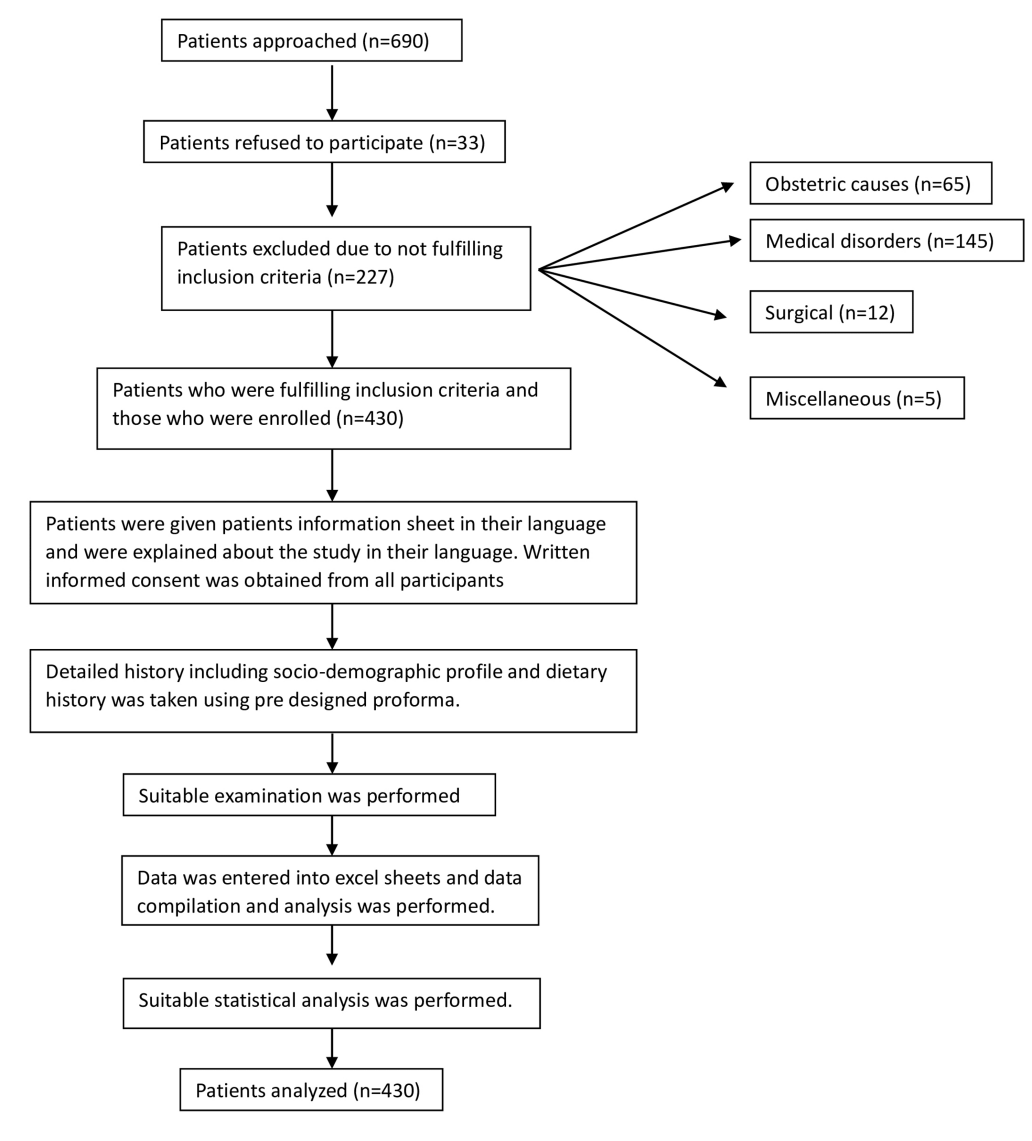
Flow chart of patients’ enrolment from antenatal clinic and methodology used.

Detailed data pertaining to sociodemographic factors, using a recall method, including the history of intake of oral iron, folic acid, and tea intake in the present pregnancy was elucidated in detail. Women who consumed iron and folic acid for more than seven days were considered for positive intake. Tea intake was considered as the number of cups of tea per day during pregnancy. Patients were enrolled as per Figure 1. Weight and height were recorded using a standard calibrated weighing scale and stadiometer and BMI was calculated. Hb levels were estimated using the HemoCue method.

A Hb concentration of less than 11 gm% was taken as the definition of anemia with further grading being mild anemia (Hb = 9-11 gm%), moderate anemia (Hb = 7-9 gm%), severe anemia (Hb = 4-7 gm%), and very severe anemia (Hb < 4 gm%).

All patients with anemia in pregnancy were randomly prescribed one tablet of 400 mg of oral albendazole for deworming in the second trimester of pregnancy. All patients with anemia were advised to consume an iron-rich diet with green leafy vegetables and fruits. Vitamin C was not routinely prescribed for anemia.

Sample size

Taking the prevalence of anemia in pregnancy in Delhi as 42% as per the NFHS-5 and using the following formula for sample size calculation: Z2 × P × (1-P); L2 (absolute error); the sample size was calculated as follows: (1.96) 2 × 0.42 (1 - 0.42); 0.05 × 0.05 = 374 was the sample size. Taking about a 10% non-response rate, the sample size was 411, which was rounded to 430.

Inclusion criteria

All pregnant women in the second and third trimesters attending the antenatal clinic were included in the study.

Exclusion criteria

Patients with a history of comorbidities, such as HIV infection, diabetes mellitus, tuberculosis, liver disease, renal disease, and malignancy, and obstetric complications like threatened abortion and antepartum hemorrhage leading to blood loss were excluded.

Written informed consent was obtained from the pregnant women after explaining to them the purpose of the study in their language and they were assured of the confidentiality and privacy of records.

Data analysis and statistical methods

Data were computerized using an Excel spreadsheet (Microsoft Corporation, Redmond, WA) and the authenticity of the data was verified. Statistical analysis was carried out using STATA version 18.0 statistical software (StataCorp LLC, College Station, TX). Categorical data were presented as frequency and percentage values. The prevalence of anemia was calculated as per the Indian standard and the association between anemia and oral iron, folic acid tablets, and tea intake was tested using the chi-square/Fisher’s exact test as appropriate. Continuous variables were tested for normality assumptions using the Kolmogorov-Smirnov test. For normally distributed data, descriptive measures such as mean, standard deviation, and range values were computed. Comparison of mean values was performed using Student's independent t-test or one-way analysis of variance (ANOVA) test as appropriate. Skewed data were presented as median and interquartile range values and compared using the Mann-Whitney U test or Kruskal-Wallis test as appropriate. For all statistical tests, a two-sided probability of p < 0.05 was considered for statistical significance.

Ethical consideration

The study was approved by the ethical committee of the institution vide approval number IEC/VMMC/SJH/Thesis/06/2022/CC-T1 (dated July 11, 2022).

## Results

**Figure 2:**
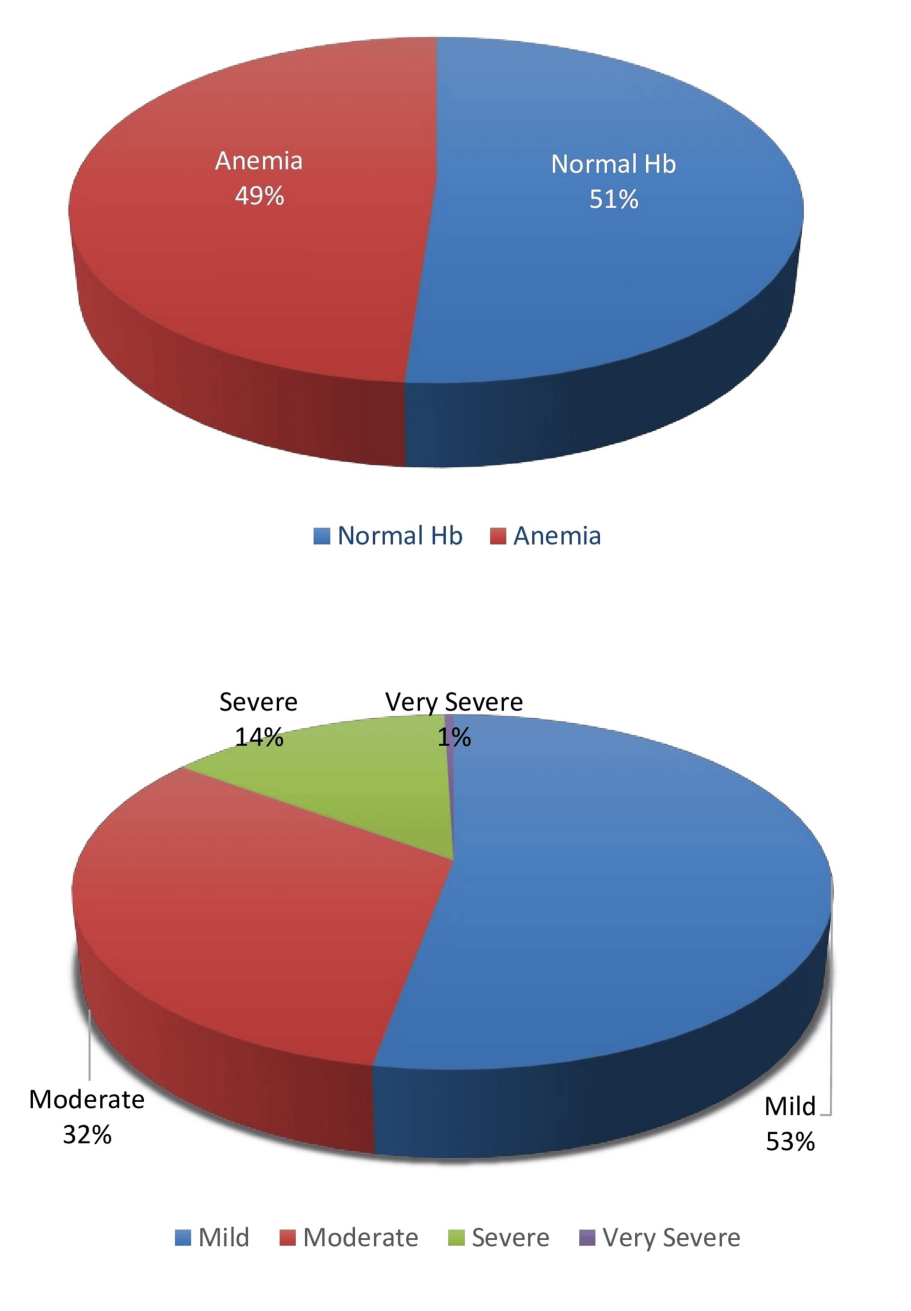
Pie charts showing the prevalence of anemia and various types of anemia as per severity. Hb: hemoglobin.

The characteristics of patients and the prevalence and severity of anemia during pregnancy are shown in Table 1 and Table 2, respectively. The mean age, parity, gestation, and BMI were 26.2 ± 4.5 years, 1.8 ± 1.2, 22.5 ± 4.5 kg/m2, and 32.2 ­± 3.4 weeks, respectively. Out of a total of 430 pregnant women, anemia was seen in 210 (48.84%) women, with mild anemia in 25.81% (52.86% of the anemia group), moderate anemia in 15.81% (32.38% of the anemia group), severe anemia in 6.98% (14.29% of the anemia group), and very severe (decompensated) anemia in only one (0.24%) case. The prevalence and severity of anemia are also depicted in pie charts in Figure 2.

**Table 1:** Characteristics of the patients (N = 430).

	Age	Parity	BMI (kg/m^2 ^)	Gestation in weeks
Range	18-43	0-5	16-35	12-14
Mean	26.2 ± 4.5 years	1.8 ± 1.2	22.5 ± 4.5	32.2 ± 3.4

**Table 2:** Prevalence and severity of anemia during pregnancy (N = 430). NA: not applicable.

	Number	Percentage out of total	Percentage out of the anemia group
Normal hemoglobin	220	51.16%	NA
Total anemia	210	48.84%	100%
Mild	111	25.81%	52.86%
Moderate	68	15.81%	32.38%
Severe	30	6.98%	14.29%
Very severe	1	0.24%	0.47%

The effect of oral folic acid tablets on the prevalence and severity of anemia is shown in Table 3. A total of 250 (58.13%) women were taking oral folic acid tablets during pregnancy, with 137 (62.27%) in the normal Hb group and 113 (53.80%) in the anemia group (p = 0.04). A total of 180 (41.86%) women did not take oral folic acid tablets during pregnancy, with 83 (37.72%) in the normal Hb group and 97 (46.19%) in the anemia group (p = 0.04). Thus overall anemia was significantly more common in women not taking oral folic acid tablets. On further break up of women not taking oral folic acid tablets, a total of 39 (35.13%) were in the mild anemia group (p = 0.12, non-significant), 34 (50%) were in the moderate anemia group (p = 0.03), and 24 (77.41%) were in the severe anemia group (p = 0.01). Hence, the non-intake of oral folic acid tablets was a risk factor for anemia in pregnancy, especially moderate and severe anemia.

**Table 3:** Effect of oral folic acid tablets intake on the prevalence and severity of anemia in pregnancy. NA: not applicable; S: significant.

Folic acid intake	Normal (n = 220)	Mild (n = 111)	Moderate (n = 68)	Severe (n = 31)	Anemia (n = 210)	Total (n = 430)	P-value	Significance
Yes	137 (62.27%)	72 (64.86%)	34 (50%)	7 (22.58%)	113 (53.80%)	250 (58.13%)	0.04	S
No	83 (37.72%)	39 (35.13%)	34 (50%)	24 (77.41%)	97 (46.19%)	180 (41.86%)	0.04	S
Total	220	111	68	31	210	430	NA	NA

The effect of oral iron tablets on the prevalence and severity of anemia in pregnancy is shown in Table 4. Only women who took oral iron for at least 30 days were considered positive for oral iron intake and those not taking or taking it irregularly and for less than 30 days were considered as non-iron intake. A total of 308 (71.62%) women were taking oral iron tablets during pregnancy, with 201 (91.36%) in the normal Hb group as compared to 107 (50.95%) in the anemia group (p = 0. 02), which was a significant difference. A total of 122 (28.37%) women were not taking oral iron tablets during pregnancy, with significantly more women (103, 49.04%) in the anemia group than in the normal Hb group (19, 8.63%) (p = 0.02). On further analysis, a total of 26 (23.42%) women were not taking oral iron tablets in the mild anemia group (p = 0.04), 48 (70.5%) were not taking oral iron tablets in the moderate anemia group (p = 0.02) while most (29, 93.55%) women were not taking oral iron tablets in the severe anemia group (p = 0.001). Hence, the non-intake of oral iron tablets was a significant risk factor for anemia in pregnancy, especially in the moderate and severe anemia group where the majority of patients did not take oral iron tablets.

**Table 4:** Effect of oral iron tablets intake on the prevalence and severity of anemia in pregnancy. NA: not applicable; S: significant.

Iron	Normal (n = 220)	Mild (n = 111)	Moderate (n = 68)	Severe (n = 31)	Anemia (n = 210)	Total (n = 430)	P-value	Significance
Yes	201 (91.36%)	85 (76.57%)	20 (29.4%)	2 (6.45%)	107 (50.95%)	308 (71.62%)	0.02	S
No	19 (8.63%)	26 (23.42%)	48 (70.5%)	29 (93.55%)	103 (49.04%)	122 (28.37%)	0.02	S
Total	220	111	68	31	210	430	NA	NA

The association of tea intake with the prevalence and severity of anemia is shown in Table 5. The history of tea intake as cups per day during pregnancy was elucidated for all patients by recall method. A total of 106 (24.65%) women were not taking tea during pregnancy with significantly more women in the normal Hb group (66, 30%) than in the anemia group (40, 19.04%) (p = 0.04). A total of 33 (7.67%) pregnant women were taking only one cup of tea per day with 19 (8.6%) in the normal Hb group and 14 (6.66%) in the anemia group (p = 0.08). The majority of women (281, 65. 34%) were consuming two cups of tea per day with significantly more women in the anemia group (147, 70%) than in the normal Hb group (134, 60.9%) (p = 0.05). On further analysis, 66 (59.45%) women in the mild anemia group (p = 0.06), 56 (82.35%) in the moderate anemia group (p = 0.03), and 25 (80.64%) (p = 0.04) in the severe anemia group were consuming two cups of tea per day. Only 10 (2.32%) women were consuming three or more cups of tea per day, and most of them (9, 4.28%) were in the anemia group than in the normal Hb group (1, 0.45%) (p = 0.05). On further analysis, one (0.9%) in the mild anemia group (p = 0.06), one (1.47%) in the moderate anemia group (p = 0.05), and four (12.90%) in the severe anemia group (p = 0.02) were consuming three or more cups of tea per day. Hence, the consumption of two or more cups of tea per day was a significant risk factor for anemia in pregnancy, especially moderate and severe anemia.

**Table 5:** Association of tea intake in cups per day on the prevalence and severity of anemia in pregnancy. NA: not applicable; NS: not significant; S: significant.

	Normal (n = 220)	Mild (n = 111)	Moderate (n = 68)	Severe (n = 31)	Anemia (n = 210)	Total (n = 430)	P-value	Significance
No tea	66 (30%)	34 (30.63%)	5 (7.35%)	1 (3.22%)	40 (19.04%)	106 (24.65%)	0.04	S
1 cup/day	19 (8.6%)	10 (9%)	3 (4.41%)	1 (3.22%)	14 (6.66%)	33 (7.67%)	0.08	NS
2 cups/day	134 (60.9%)	66 (59.45%)	56 (82.35%)	25 (80.64%)	147 (70%)	281 (65.34%)	0.05	S
3 or more cups/day	1 (0.45%)	1 (0.9%)	1 (1.47%)	4 (12.90%)	9 (4.28%)	10 (2.32%)	0.05	S
Total	220	111	68	31	210	430	NA	NA

## Discussion

Anemia is a widespread global health problem affecting about 1.5 billion people with higher prevalence in Asia and Africa [1,3]. India has one of the highest prevalence of anemia in pregnancy with older studies showing prevalence between 47% and 96% [4]. The overall prevalence of anemia during pregnancy in the present study was 210 (48.84%) (25.81% with mild anemia, 15.81% with moderate anemia, 6.98% with severe anemia, and 0.24% with very severe anemia), which was lesser than the prevalence of anemia during pregnancy in NFHS-5 (2019-2021) (52.2%) but was more than that of Delhi (42.2%) (NFHS-5) [3]. It could be due to the study being done in a referral public hospital in Delhi, which caters to low-income group patients of Delhi and offers free treatment. In the present study, a total of 41.86% of patients did not take oral folic acid tablets during pregnancy, with a significantly higher prevalence of anemia in them, thus making non-intake of oral folic acid tablets a risk factor for anemia in pregnancy. Similarly, in the present study, a total of 122 (28.37%) patients did not take oral iron tablets and anemia was significantly more in them. The difference was even more significant for moderate anemia and severe anemia groups where 48 (70.5%) and 29 (93.55%) patients, respectively, did not take oral iron tablets. Hence non-intake of oral iron tablets during pregnancy was a risk factor for anemia in pregnancy being an even bigger risk factor for moderate and severe anemia. The results of the present study are similar to other national and international studies. Abd Rahman et al. [12] from Malaysia observed non-compliance to hematinics as a risk factor for anemia in pregnancy. The intake of oral folic acid tablets in the present study was 250 (58.13%) and the intake of oral iron tablets was 308 (71.62%), which was much higher than other previous studies. It could be due to the fact that other studies were done in the past while the present study was performed in 2023 in the capital city of Delhi with more awareness and understanding about the consumption of oral folic acid and iron tablets in pregnancy. Also, the present study was conducted on patients attending the antenatal clinic of a tertiary hospital where in routine women are advised to consume oral folic acid and iron tablets during pregnancy and are given free tablets from the hospital pharmacy. The higher percentage of oral iron tablets (308, 71.62%) than oral folic acid tablets (250, 58.13%) could be because most patients came for booking in the second or third trimester when iron tablets with folic acid are prescribed.

Ali et al. [13] from Unguja Island, Tanzania (Africa) also observed poor compliance or non-intake of oral iron and folic acid supplementation as a risk factor for pregnancy anemia.

Tea intake, especially two or more cups per day, was associated with a higher prevalence of anemia during pregnancy in the present study. Our results are similar to Zhang et al. [14], who observed tea or coffee intake, especially after meals, as a risk factor for anemia in pregnancy in their study from China. Sheriff et al. [15] also observed a higher prevalence of anemia in pregnancy in the tea state community of Sri Lanka in their study. Zewar and Chakraborty [16] from Afghanistan also observed tea or coffee intake as a risk factor for anemia in pregnancy in their study. The effect of tea or coffee is due to the presence of tannin in them, which decreases the absorption of iron from the gastrointestinal tract, thus decreasing the bioavailability of oral iron.

Joe et al. [17] studied the coverage of iron and folic acid supplementation in India under the Anemia Mukt Bharat program and observed an increase in coverage of iron and folic acid supplementation from 78% in 2017-2018 to 90% in 2019-2020. However, it did not decrease the prevalence of anemia in pregnancy. Proper counseling of patients, family attention to ensure adherence to iron and folate supplementation and food fortification, and routine oral iron and folic acid supplementation can play a great role in reducing the prevalence of anemia [18-21]. Oral iron preparations are the first choice but prenatal iron can be given in suitable cases [22]. Enhancing access to maternal health services, antenatal care, and contraception, and improved sanitation facilities would supplement the existing interventions targeted to reduce anemia [23]. In an Indian study by Srivastava et al. [24] conducted on 204 pregnant women who were either prescribed iron capsules or iron tablets, intake of iron capsules resulted in higher compliance but an equal rise in mean Hb and ferritin as compared to iron tablets. In another community-based study by Ahamed et al. [25] in Haryana, India, iron and folic acid were given under dietary supervision in the study group and without supervision in the control group. Another Indian study by Singh et al. [26] provided health education to all pregnant women to consume iron folic acid tablets but only 42.31% of patients consumed oral iron tablets for more than 90 days. Strengthening the need for constant motivation of antenatal women by healthcare workers. Despite the National Nutritional Anemia Prophylaxis Programme (NAAPP) [27] initiated in 1970 as a measure to prevent anemia in the country, the prevalence of anemia did not decrease much. The Anemia Mukt Bharat [28] strategy was started by the Government of India in 2019 to reduce anemia among six beneficiary groups, including pregnant women, using six interventions out of which prophylactic iron and folic acid supplementation of Anemia Mukt Bharat should decrease the prevalence of anemia in all beneficiaries, including pregnant and lactating women.

## Conclusions

The result of the present study shows that anemia during pregnancy remains a public health issue. Despite the free availability of oral iron and folic acid supplementation, 180 (41.86%) patients did not take oral folic acid tablets, and 122 (28.37%) patients did not take oral iron tablets. There is a need for counseling of pregnant women and families for the consumption of oral folic acid and iron tablets.

Anemia is a multifactorial disease with many factors playing a role in its etiology. The present study was a cross-sectional study on pregnant women in the second and third trimesters of pregnancy attending the antenatal clinic of a tertiary referral health hospital that provides free treatment and care to poor patients. The prevalence is not a true representative of the community and is more hospital-based. Also, it was a one-time study with no follow-up and based on recall with the risk of missing some data and recall bias.
